# Early embryonic determination of the sexual dimorphism in segment number in geophilomorph centipedes

**DOI:** 10.1186/2041-9139-4-22

**Published:** 2013-08-06

**Authors:** Carlo Brena, Jack Green, Michael Akam

**Affiliations:** 1 Laboratory for Development and Evolution, Department of Zoology, University of Cambridge, Downing Street, Cambridge CB2 3EJ, UK

**Keywords:** Segmentation, Sex determination, Intraspecific variability, *Strigamia maritima*, Molecular sexing assay

## Abstract

**Background:**

Most geophilomorph centipedes show intraspecific variability in the number of leg-bearing segments. This intraspecific variability generally has a component that is related to sex, with females having on average more segments than males. Neither the developmental basis nor the adaptive role of this dimorphism is known.

**Results:**

To determine when this sexual dimorphism in segment number is established, we have followed the development of *Strigamia maritima* embryos from the onset of segmentation to the first post-embryonic stage where we could determine the sex morphologically. We find that males and females differ in segment number by Stage 6.1, a point during embryogenesis when segment addition pauses while the embryo undergoes large-scale movements. We have confirmed this pattern by establishing a molecular method to determine the sex of single embryos, utilising duplex PCR amplification for Y chromosomal and autosomal sequences. This confirms that male embryos have a modal number of 43 segments visible at Stage 6, while females have 45. In our *Strigamia* population, adult males have a modal number of 47 leg-bearing segments, and females have 49. This implies that the sexual dimorphism in segment number is determined before the addition of the last leg-bearing segments and the terminal genital segments.

**Conclusions:**

Sexual dimorphism in segment number is not associated with terminal segment differentiation, but must instead be related to some earlier process during segment patterning. The dimorphism may be associated with a difference in the rate and/or duration of segment addition during the main phase of rapid segment addition that precedes embryonic Stage 6. This suggests that the adaptive role, if any, of the dimorphism is likely to be related to segment number *per se*, and not to sexual differentiation of the terminal region.

## Background

Centipedes of the Order Geophilomorpha provide attractive models for studying the specification and evolution of segment number in arthropods. They show extreme variability in adult segment number, with the number of leg-bearing segments ranging from 27 to 191 between species [1]. This number is always odd – a striking example of constraint in natural variation [2]. All of these segments develop during embryogenesis: juveniles hatch with the final adult number of leg-bearing segments already present. In this respect, geophilomorphs are derived among centipedes, sharing this trait only with their sister group the Scolopendromorpha.

Most species show intraspecific variation in segment number. In our study species, *Strigamia maritima*, segment numbers range from 43 to 53 in the UK population. Several factors are known to influence this number. There is geographic variation between populations [3], probably due at least in part to the influence of temperature during embryogenesis [4,5]. There is also an influence of genetics within populations [6], and possibly also between populations. Most significantly for this work, there is also an influence of sex. In *Strigamia*, females of any given population typically have a modal number of segments that is two higher than males. Similar patterns are observed in most species of the order, with the sexes differing by two segments in many forms, but exceptionally by as many as 16 segments in some of the species with the largest number of segments [7]. Within the order, only one basal group, the Mecistocephalidae, do not generally show such a difference between the sexes [8].

**Figure 1:**
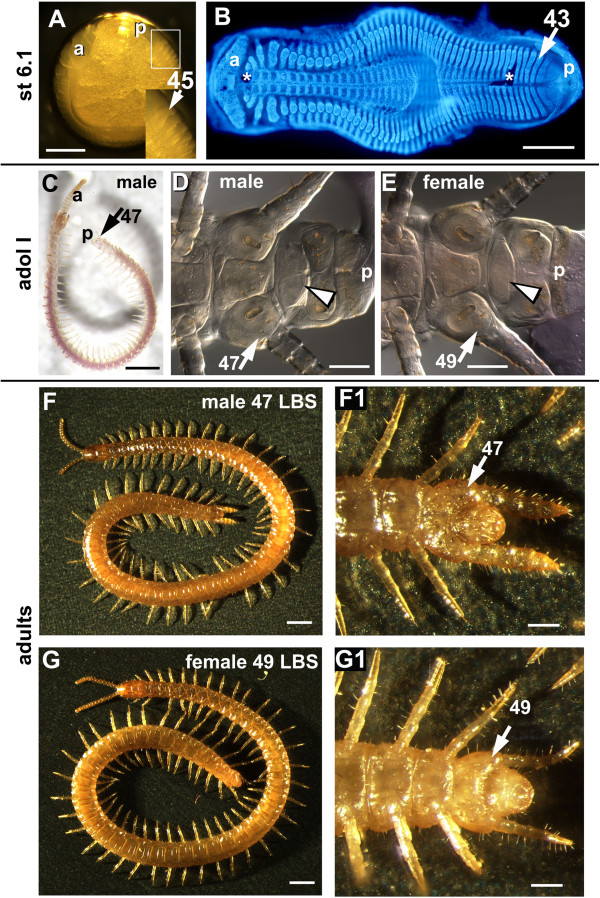
***Strigamia *embryonic and post**-**embryonic stages used for leg-bearing segment counting and sex determination. (A)** Lateral view of an early Stage 6 embryo, live in mineral oil: lateral illumination and black background allow segment counting (45 leg-bearing segments (LBSs) in this specimen, see insert). **(B)** An early Stage 6 flat-mounted germ band, stained with DAPI (4^′^,6-diamidino-2-phenylindole) to highlight the morphology. The stage of this specimen corresponds to that of the embryo shown in **(A)** and is characterised by the lateral spreading of the germ band in its middle portion. This specimen has 43 LBSs; the 44th LBS here begins to appear, but would not be visible in the live embryo. **(C)**, **(D)**, **(E)** Adolescens I stage: **(C)** live specimen in mineral oil, lateral view; **(D)**, **(E)** high magnification of the ventral side of the terminal trunk mounted on a slide and viewed at the compound microscope with Nomarski optics. This is the first stage at which it is possible to determine the sex because of the sexual differentiation of the genital sternite (arrowhead), with lateral protrusions in males **(D)** and rounded in females **(E)**. At this stage, the legs of the last LBS are already distinct, notably by the swollen coxopleuron, but they are not obviously different in males and females. **(F)**, **(G)** Final differentiation between live adult male and female: in the studied population, the modal number of LBSs in males is 47 **(F)** and in females is 49 **(G)**. By this stage the last pair of legs are highly modified in the male, swollen and bearing sensory and glandular structures **(F,F1)**. **(F)**, **(G)** dorsal view; **(F1)**, **(G1)** ventral magnified view of, respectively, **(F)** and **(G)**. a, antenna; p, proctodeum.*Artefactual rupture of the germ band. Scale bar: **(A)**, **(B)** 300 μm; **(C)** 1mm; **(D)**, **(E)** 100 μm; **(F)**, **(G)** 1 mm; **(F1)**, **(G1)** 300 μm.

We can consider two general models for the developmental origin of this sexual dimorphism in segment number. One model, which we initially considered the most likely, focuses on the fact that the genital segments of centipedes lie at the extreme posterior end of the body. The last pair of leg-bearing segments is strikingly dimorphic in many species (see, for example, Figure 1F,G), and behind this there are highly modified segments associated with the gonopods, gonopores and egg-laying apparatus. Little is known about the development of these genital segments, which are not developed at hatching. It seemed possible that different numbers of segments may be specified according to sex in this extreme posterior region, or that segment primordia might develop differentially in the two sexes, such that two segments which in females develop as leg-bearing segments might make no appendages, or specialised genital structures, in males. Something similar is known to happen in *Drosophila* and some other Diptera, where male and female external genitalia develop from different segmental primordia [9,10].

A second class of model proposes that the intrinsic mechanisms of segment patterning, and in particular segment counting, are different in males and females. In embryos of both sexes, a characteristic and invariant set of six head segments are first generated, followed by the poison claw or forcipular segment. Structurally the poison claw forms part of the head, but this segment is generally considered to be the highly modified first segment of the trunk. Leg-bearing segments are added in sequence behind the poison claw. These are broadly similar to one another, although they differ in size and in some details of patterning. In *S. maritima*, most of the leg-bearing segments are added at a constant rate of about one segment every 3 hours (at 13°C) during Stage 4 [11]. They appear singly in sequence from front to back, although molecular markers show that a prepattern of double segment periodicity precedes definitive segment patterning. This prepattern involves dynamic gene expression that suggests the existence of a segmentation oscillator akin to that described in vertebrates [12,13]. We have previously suggested that variation in segment number might depend on the number of cycles undergone by this oscillator, and hence the number of double segment units initially generated [12,13]. Hence one model for sexual dimorphism in segment number might suggest that sex influences the number of cycles undergone by this oscillator.

Segment addition slows markedly during Stage 5, before pausing completely at Stage 6, when the embryo undergoes a dramatic movement, in which the left and right sides of the germ band separate before flexing sharply and sinking into the yolk (Figure 1A,B). Segment addition resumes slowly after sinking, with the last few leg-bearing segments appearing before hatching [11]. The mechanisms that pattern these last segments appear to differ somewhat from those involved during the major rapid phase of segment addition (C Brena and M Akam, in preparation).

At hatching, the most posterior segments are poorly differentiated – the final leg-bearing segment is defined only by a segmental groove that appears after apolysis of the embryonic cuticle; it does not have a limb bud at this stage [14] (C Brena, unpublished data). During the next 2 months, the hatchling undergoes a number of further moults, nourished by remaining yolk in its gut. The most posterior segments differentiate at this time, until, with the moult to the first free-living stage, adolescens I, the final pair of legs become functional and the genital segments differentiate to the point where male and female become distinguishable (Figure 1C,D,E) [5,15].

While observing the development and segmentation of living *Strigamia* embryos, one of us (CB) observed that the number of segments visible in embryos at Stage 6, when segment addition pauses, varies between 41 and 47, with a large proportion of the embryos having either 43 or 45 visible segments (counting as segment number 1 the first leg-bearing segment, immediately following the distinctive forcipular segment). This parallels the variation seen in *Strigamia* adults of this population, the majority of which have 47 leg-bearing segments in males and 49 in females. This led us to wonder whether the dimorphism in segment number might already be apparent at the time when segment addition pauses, before addition of the terminal trunk segments. If this were the case, it would favour models of Class 2 over those of Class 1 above.

Two technical developments have made it possible to test this hypothesis. First, the finding that embryos can be reared to the adolescens I stage in the laboratory, when they can be sexed, by culturing them under oil [5]; and second, the identification of male-specific sequences in the *Strigamia* genome, which allowed us to develop a molecular assay to sex single embryos. We use these methods below to show that embryos do indeed already have a clear sexual dimorphism in the number of segments patterned when segment addition pauses at Stage 6.

## Methods

*S. maritima* eggs were collected from a previously studied population near Brora (Scotland). Eggs collected in June 2009 were used for a long-term culture experiment that provided the initial observations described below. Eggs collected in June 2012 were used for the embryo sexing experiment. Individual clutches of eggs were taken to the laboratory, and cultured at 13°C either on moistened filter papers (for the embryo sexing experiment) or under a shallow layer of mineral oil, for long-term culture, as described in Brena and Akam [11]. In the latter conditions, *Strigamia* eggs can develop, hatch and develop further to the adolescens I post-embryonic stage, more than 2 months after collection [5]. Male and female adults from the same population were collected for DNA extraction.

### Embryo handling and staging

For the long-term culture experiment, individual eggs from clutches that had not yet begun segmentation were monitored, generally daily, as described by Brena and Akam [11], recording the number of the last segment morphologically defined by both anterior and posterior segmental grooves. In particular, the number of leg-bearing segments was counted from photographs of all specimens at Stage 6.1, a stage easily identified by the slight flattening of the germ band away from the chorion at its midpoint (see Figure 1A). After hatching and maturation to the adolescens I stage, surviving animals were scored for sex and segment number.

For the embryo sexing experiment, clutches of eggs were monitored over a period of weeks until they reached Stage 6. Embryos at Stage 6.1 were mounted in locust embryo saline in agarose wells, photographed in lateral view, and then snap frozen in liquid nitrogen and stored at −80°C. In both experiments, segments were counted blind, without knowing the sex of the embryo.

Pictures of whole eggs, adolescens I specimens and adults were taken with a Leica MZFLIII stereomicroscope with a Leica DFC 500 Camera (Leica Firecam software; Leica Microsystems, Cambridge, UK), using lateral light and, generally, a black background. Flat-mounted preparations – either fixed germ bands or unfixed adolescens I specimens – were photographed with a Zeiss Axiophot compound microscope (Zeiss, Jena, Germany) with a Leica FC3 FX camera. The contrast and colour of the photographs were adjusted using Adobe Photoshop. Stacks of images at multiple focal depths were combined with Helicon Focus (Helicon Soft Ltd., Kharkov, Ukraine). The flat-mounted embryo in Figure 1B was prepared and photographed as described in [11].

### Embryo sexing assay

DNA was extracted from single frozen embryos using a protocol based on that of Truett and colleagues [16]. 75 μl alkaline lysis buffer (25 mM sodium hydroxide, 0.2 mM EDTA - ethylenediamine tetraacetic acid - pH unadjusted) was added to individual embryos in microfuge tubes, and the tissue was disrupted and homogenised using a sterile pestle. The lysate was incubated at 98°C for 30 minutes, neutralised with 75 μl of 40 mM Tris–HCl, pH5, and centrifuged for 2 minutes at 6,000 rpm. The sample was then diluted 1/100 in double-distilled water, and 2 μl were used as a PCR template in each reaction.

**Figure 2:**
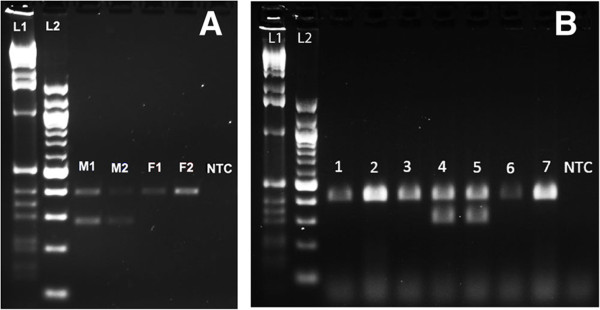
**Duplex PCR assay for sex determination in *Strigamia maritima. *(A)** Positive control. Sex-determination assay on adults of known sex. Two males, M1 and M2 generate PCR bands at 398 bp and 288 bp corresponding to the autosomal band and Y chromosome band, respectively. Two females, F1 and F2 generate a single band at 398 bp corresponding to the autosomal band. **(B)** Experimental assay. Sex determination of single embryos of unknown sex. Lanes 1, 2, 3, 6 and 7 generate single PCR bands (398 bp) and so are inferred to contain DNA derived from female embryos. Lanes 4 and 5 generate two PCR bands (398 bp, 288 bp) and so are inferred to be from males. NTC, no template control (generates no bands). The sizes of a 1 kb (L1) and 100 bp (L2) DNA ladder are shown.

For the sex-determination PCR, two sets of primers were used in each reaction. The first primer set amplifies a 398 bp region of genomic scaffold 7180001247533 (Smar genome assembly 1.0 [17]), which from its representation in the genomic sequence reads is inferred to be autosomal, and which amplifies in both male and female adults (Figure 2A). The second primer set amplifies a 288 bp region of genomic DNA from scaffold 7180001247258, which is underrepresented in the genomic sequence reads, and which is inferred to be Y chromosomal (J Green and colleagues, in preparation). This set amplifies only in adult males (Figure 2A). Male samples thus generate two PCR bands of distinct sizes, whereas female samples generate only a single PCR band (Figure 2).

PCR reactions were run with all four primers in equimolar ratios, and under the following conditions: an initial denaturation step of 95°C for 10 minutes; followed by 10 touchdown cycles, the first being 95°C for 20 seconds, 60°C for 30 seconds, 72°C for 45 seconds, in which the annealing temperature is decreased by 1°C every cycle; followed by 25 cycles of 95°C for 20 seconds, 50°C for 30 seconds, 72°C for 45 seconds; and finally an extension step of 72°C for 4 minutes. Bands were visualised on 1.5% agarose gel with 0.1 ng/μl ethidium bromide.

## Results

To determine whether the variation in segment number at Stage 6.1 is related to the sex of the embryo, a sample of 22 eggs that had been scored for segment number throughout embryogenesis [11] were maintained in culture until the adolescens I stage, the first stage at which it becomes possible to sex individuals with some degree of accuracy by morphological criteria (Figure 1D,E) [5].

**Figure 3:**
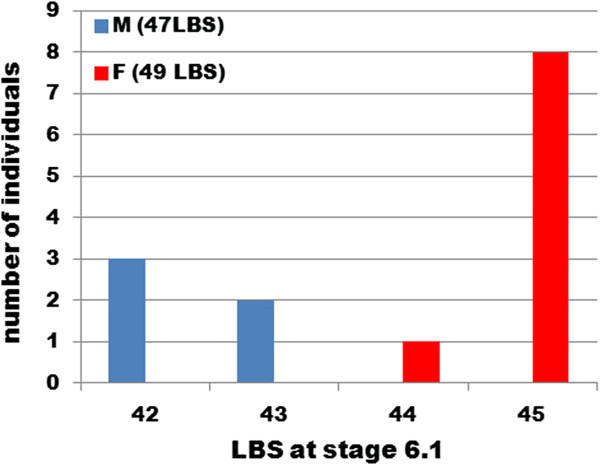
**Number of leg**-**bearing segments at embryonic stage 6.1, for individuals sexed after hatching.** Live embryos were scored for leg-bearing segment (LBS) number at embryonic Stage 6.1, reared to the adolescens I stage, and sexed morphologically. M, males (here all with 47 LBSs at the adolescens I stage); F, females (here all with 49 LBSs).

The survival of embryos through the several post-embryonic moults that precede the adolescens I stage is greatly improved by culturing them under oil, but, even so, only 14 of the 22 individuals initially scored as embryos survived the 2 months in culture to the point at which they could be sexed. However, these surviving individuals showed a very clear relationship between the number of segments at mid-embryogenesis and the sex of the individual: all males had 42 or 43 segments visible at Stage 6.1, while all females had 44 or 45 segments (Figure 3). Note that embryonic segments appear singly, at least when scored morphologically, and therefore animals are scored with both odd and even numbers of segments, in contrast to the final adult number of leg-bearing segments, which is always odd. This final number is already established in adolescens I individuals. For the animals scored here, all males showed 47 leg-bearing segments and all females show 49.

This result suggested that the dimorphism in adult segment number is established before final segment addition, but the number of surviving animals successfully sexed is too small to feel confident of this result.

Therefore, to confirm this result, we made use of the finding that the *S. maritima* genome contains male-specific DNA sequences (presumably located on a Y chromosome, but this has yet to be shown). This allowed us to develop a single embryo sexing protocol. This protocol utilises a duplex PCR reaction, with one pair of primers amplifying a genomic sequence previously shown to be present at similar copy number in male and female adults, and a second pair of primers that amplify a fragment only in adult males, not adult females (J Green and M Akam, unpublished data). The specificity of this reaction is shown for adults in Figure 2A.

A sample of embryos from 13 different clutches were reared in the laboratory until individual embryos reached Stage 6.1, when individual embryos were photographed in lateral view to allow counting of segment number, and then frozen for DNA isolation. Embryos were then sexed using the duplex PCR assay.

**Figure 4:**
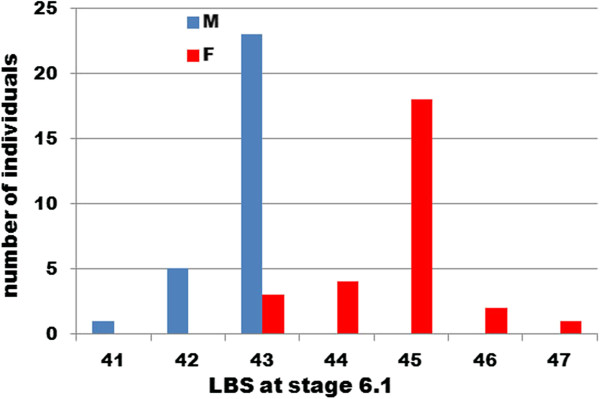
**Number of leg**-**bearing segments in embryos sexed by the molecular sexing assay.** Embryos at Stage 6.1 were photographed for segment counting and then processed by DNA extraction for the duplex PCR assay. F, females; M, males.

Of 57 embryos assayed, 29 were identified as male and 28 as female. The distribution of segment numbers scored in the two sexes is almost nonoverlapping: males showed a modal number of 43 segments, while females showed a modal number of 45 segments (Figure 4). These two distributions are different with very high probability (*P* = 6.1 × 10^–11^; paired two-tailed *t* test). This observation confirms that the dimorphism in segment number is already apparent at the mid-embryonic pause in segment addition.

Note that the distributions of segment numbers in our two experiments – the long-term culture and the embryo sexing experiments – are not expected to be precisely comparable. We know from previous work that the final adult segment number is affected by the temperature that an embryo experiences during early embryogenesis, before the start of trunk segmentation [5]. Presumably the segment number at mid-embryogenesis is also likely to be affected. Many of these eggs will have passed part or all of this temperature-sensitive period on the beach before collection, or in temperature conditions that were not well controlled while the embryos were returned to the laboratory before the start of the experiment. As the experiments were conducted in different years, these early effects of temperature will not be consistent between years, or indeed necessarily between clutches at different locations. We might therefore expect different experiments, and different clutches within experiments, to show different modal numbers of segments. However, all embryos of a single clutch will have followed very similar temperature profiles, and so far as we know eggs within a single clutch are equally likely to be male or female. There should therefore be no consistent bias within the individual experiments.

Despite this uncertainty, it is notable that, if we pool the data for both of our experiments, the average segment numbers estimated in males and females at Stage 6.1 differ by 2.1 segments (means: 42.7 male, *n* =34; 44.8 female, *n* =37), whereas for adults in the Brora population the difference was estimated as 2.2 segments (means: 46.7 male, *n* =72; 48.9 female, *n* =72) by Kettle and Arthur [3]. Most if not all of the divergence in segment number is thus already apparent at mid-embryogenesis.

**Figure 5:**
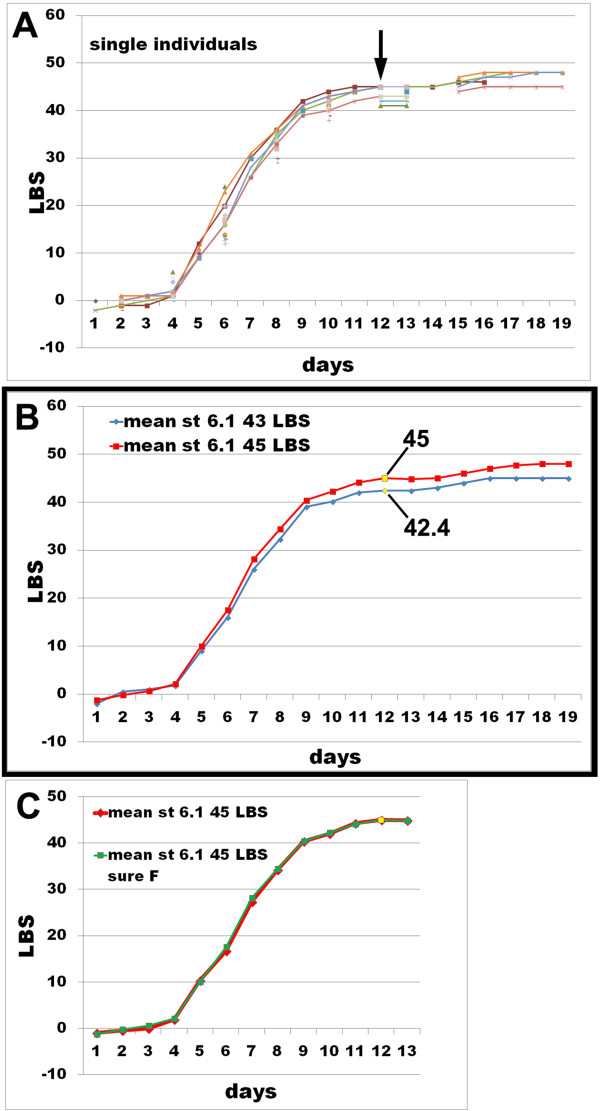
**Rate of addition of leg**-**bearing segments through embryonic development. (A)** Plots show the segment number each day for single embryos (each represented by a different colour) (*n* = 22); interrupted or fragmentary lines correspond to embryos for which sure daily data were not available. **(B)** Plots show the daily averaged segment number for the embryos of **(A)** having a leg-bearing segment (LBS) number value of 41 to 43 at Stage 6.1 (blue line, ‘mean st 6.1 43 LBS’) (*n* =14) and for the embryos of **(A)** having a LBS number value of 44 to 45 (red line, ‘mean st 6.1 45 LBS’) (*n* =8). **(C)** Plot comparing the red line of **(B)** (‘mean st 6.1 45 LBS’) with a selection (*n* =9) of those embryos whose sex has been determined at stage adolescens I. The plot shows that the trend of the daily mean values of **(B)** is a precise representation of the trend of known female individuals, with an adult LBS number of 49. LBS values at Stage 6.1 are marked by an arrow in **(A)** and are in yellow in **(B)** and **(C)** (specific values indicated in **(B)**). Negative LBS values indicate anterior, nonleg-bearing segments: 2, first maxilla segment; –1, second maxilla segment; 0, maxilliped segment.

In the long-term culture experiment, the available data for animals that survived to adolescens I suggest that embryos scored as having 42 or 43 segments at Stage 6.1 are likely to be males, while embryos scored with 44 or 45 segments at this stage are likely to be females. If we use this criterion to assign sex, regardless of whether the individual survived long enough to be sexed, we can plot the trajectory of segment addition by sex, averaged on a daily basis for all individuals in the dataset (Figure 5B). This plot shows that putative males and females appear to differ slightly in the rate of segment addition during the rapid phase of trunk segment addition (Stage 4), but that from Stage 6 onwards the segment numbers of males and females at the same age maintain a constant difference of about two in segment number. If the data are averaged only for those females for which sex is definitively known, the plot is virtually identical to that for putative females in the entire dataset (Figure 5C). For males, too few sexed individuals are available to make this comparison meaningful.

Note however, that in this data set sex is absolutely correlated with final segment number, so we cannot distinguish between a difference that relates to sex and a difference that relates to final segment number, independent of sex. To do this would require growing a population under temperature conditions such that at least one sex produces a workable proportion of embryos with differing segment numbers.

In fact, there is appreciable variability in the recorded trajectories for individual embryos (Figure 5A), probably at least in part because embryos were only scored at 24-hour intervals, with the result that the synchrony of sampled points in relation to developmental stage is not precise. While the averaged plot is suggestive, we do not believe that it can be taken to discriminate between a model in which it is the rate of segment addition that varies between embryos, and one in which it is the duration of the segment addition phase that accounts for the final difference in segment number between males and females. To do this would require more frequent sampling of the segment number, to determine more precisely the time of onset of segment addition in individual embryos. Unfortunately, with our current experimental procedures, the more frequently embryos are observed, the more likely they are to show abnormal development, precluding such observations (see [11]).

## Discussion

Our data show clearly that the sexual dimorphism in segment number in *Strigamia* is established by the time segment addition pauses, about three-quarters of the way through embryogenesis, and before the last few leg-bearing segments have been added. Therefore this dimorphism can have nothing to do with the patterning or differentiation of the genital segments, which lie terminal to the leg-bearing segments, and do not develop until after hatching: the same number of terminal leg-bearing segments appears to be added after Stage 6 in both males and females.

This is in marked contrast to the situation in *Drosophila* and other derived Diptera, where both sexes specify the same number of segment primordia initially, and the adult difference arises subsequently through the differential development of these primordia [9,10].

There is no overt sexual differentiation of centipede embryos at Stage 6, besides the evident difference in segment number. This suggests that the mechanism regulating the difference in segment number may relate not to any aspect of functional sexual differentiation, but to some intrinsic difference in the behaviour of male and female cells during the process of development before and during segmentation. This might, for example, affect the rate of cell replication or segment addition, and/or the number of cells allocated to each segment.

Recent work in *Drosophila* has suggested one possible mechanism through which sex might influence segmentation. Manu and colleagues have shown that one of the key genes in the *Drosophila* segmentation cascade, *evenskipped*, is expressed differentially in males and females [18]. They attribute this difference to incomplete dosage compensation of an X-linked upstream regulatory gene, *giant*, which represses *eve*. In *Drosophila*, this difference in *eve* expression does not lead to any persistent difference in final segment patterning, but in the long-bodied centipedes with far more segments such a subtle difference in gene regulation, due to gene dosage effects, might have persistent effects. For example, if on average the difference resulted in one more cycle of the putative segmentation oscillator before segment addition pauses at Stage 6, this would account for the modal difference in segment number between males and females. (Note that this early patterning process generates double segment units, each of which is then subdivided to make a pair of individual segments [12,13]).

Segment number in *Strigamia* depends not only on the sex of the embryo, but also on the temperature at which the embryo develops. At high temperatures, embryos develop more segments [4]. This is not due to an effect on the sex ratio of the population, and the effect is seen equally in embryos of both sexes, so the effect of temperature on segment number cannot be mediated by biasing the sex ratio [5]. The development of more segments must therefore depend on some effect of temperature on the control of segment number itself.

The sensitive period for this effect of temperature is well before Stage 6, and indeed before the majority of trunk segments have been patterned: once the first trunk segments are morphologically visible, temperature has little if any effect on final segment number [5]. Although there is no necessary connection between the effects of sex and temperature on segment number, it may be that these two parameters are affecting the same very early process – for example, the number of cells present at the time when trunk segmentation initiates.

Unfortunately, in the absence of techniques to visualise the behaviour of individual cells in living embryos, and given the difficulty of obtaining populations of *Strigamia* embryos of precisely similar age, it will not be easy to discriminate between these various hypotheses at present, particularly as the difference in segment number between males and females is only some 4%, and so could be accounted for by a very small difference in cell behaviour. If the number of cells allocated to each segment differs by about this magnitude, however, this should in principle be detectable.

Among geophilomorph centipedes there is a close correlation between those species that show a sexual dimorphism in segment number, and those that show variation in segment number between individuals of the same sex. In the most speciose suborder of the geophilomorphs, the Adesmata, most if not all species show this pattern of variability. In the basal sister group to the Adesmata, the Mecistocephalidae, only few species show inter-individual variation in segment number, and these may also show sexual dimorphism, although this is not certain [8,19,20]. These observations suggest that there may be a link between the mechanisms underlying individual variation and sexual dimorphism in segment number.

## Conclusion

In *S. maritima*, and probably by extension in other geophilomorph centipedes, the sexual dimorphism in segment number is established before the addition of the final leg-bearing segments, and well before the appearance of the terminal region that bears the genitalia. It is therefore unlikely to have anything to do with sexual differentiation in the genital region, and more likely to be a result of some effect of sex on the developmental mechanism that counts segments.

This raises the question of whether the difference in segment number between males and females is of adaptive significance, or whether it is an incidental side effect of the developmental process in the two sexes that is simply tolerated by selection. The answer is unknown. If the sexual dimorphism is adaptive, however, its role is more likely to be related to segment number *per se* than to sexual differentiation of the terminal region.

## Abbreviations

Bp: Base pair; PCR: Polymerase chain reaction.

## Competing interests

The authors declare that they have no competing interests.

## Authors’ contributions

CB developed the long-term culture assay and first proposed the hypothesis that sexual dimorphism in segment number is established before final segment addition. JG identified sex-specific genomic sequences and developed the embryo sexing assay. MA proposed the duplex PCR assay. All authors participated in the drafting of the text, and all read and approved the final manuscript.
